# Safety and Efficacy of Polymethylmethacrylate-Collagen Gel Filler for Correction of the Pre-jowl Sulcus: A 24-month Prospective Study

**DOI:** 10.1093/asjof/ojac030

**Published:** 2022-04-22

**Authors:** Oscar Hevia

## Abstract

**Background:**

Polymethylmethacrylate (PMMA)-collagen gel is approved for the correction of nasolabial folds and severe atrophic, distensible facial acne scars on the cheek. However, fillers are often used off-label in clinical practice, necessitating additional study of safety and efficacy.

**Objectives:**

To determine the safety and efficacy of PMMA-collagen gel for the correction of lower face aging, specifically the pre-jowl sulcus.

**Methods:**

In this prospective, single-center, 1-year study (N = 20) and additional 1-year extension (N = 10), 20 patients with a pretreatment score of 2, 3, or 4 on the 5-point Merz Aesthetic Scale for jawline at rest were eligible for treatment with PMMA-collagen gel. Efficacy was measured by blinded review using the jawline scale, Subject and Physician Global Aesthetic Improvement Scale (GAIS) and Subject Satisfaction scores, collected at weeks 4, 12, 26, 52, and 104.

**Results:**

Improvement in jawline score was significant at all posttreatment time points up to 104 weeks (*P* < 0.01). The percentage of patients with subject-reported GAIS ratings of “improved” or “much improved” was 79% at 12 weeks and ratings were maintained at 76% at 52 weeks and increased to 90% at week 104. At 52 and 104 weeks, 82% and 100% of patients, respectively, were at least “somewhat satisfied.” All adverse events were minor.

**Conclusions:**

PMMA-collagen gel is well tolerated and effective for durable correction of the pre-jowl sulcus and jawline.

**Level of Evidence: 4:**

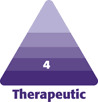

Volume loss in the lower face due to worsening tissue laxity, bone resorption, fat atrophy, and laxity of the mandibular septum results in a less-defined, sagging jawline as well as the development of melomental folds and jowls.^1-3^ Dermal fillers can provide a significant, nonsurgical treatment option by filling, lifting, and/or redefining many of these aging landmarks. The lower face represents a large treatment area, which, in many cases, requires an extensive volume of filler to correct. This requirement subjects the patient to considerable treatment cost and a more intensive injection experience (greater number of treatments and syringes needed). Using a long-lasting filler in this anatomical area may alleviate the repetitive nature of treatment with less durable fillers. For this reason, polymethylmethacrylate (PMMA)-collagen gel (Bellafill, Suneva Medical, Inc., San Diego, CA) is a reasonable choice to consider and present to the patient. PMMA microspheres are not absorbed by the body, and once injected stimulate ongoing neocollagenesis, the physiochemical basis for its longevity.^4^ This property translates into clinical durability as well. In a previous, multicenter study, 84% of patients treated in the nasolabial folds were satisfied/very satisfied at the end of the 5-year study.^5^

Though PMMA-collagen gel is approved for the treatment of acne scars and nasolabial folds,^6,7^ it is commonly used off-label to treat areas such as the cheek, temple, chin, and jawline.^8^ This expanded use necessitates additional studies to evaluate the safety and efficacy of PMMA-collagen gel in these anatomical areas, in particular for clarifying expected outcomes, optimal injection technique, and patient selection. In this study, the safety and efficacy of PMMA-collagen gel were examined for correction of lower face aging, specifically the pre-jowl sulcus. Results of a 1-year study and additional 1-year extension study are presented here.

## METHODS

### Study Design

In this 1-year, single-center, single injector, prospective study with 1-year extension, 20 patients (ages 21-75 years) with grade 2-4 sagging on the left and right jawline on the validated, 5-point (0-4) Merz Aesthetic Scale for jawline at rest (0 = no sagging, 1 = mild sagging, 2 = moderate sagging, 3 = severe sagging, and 4 = very severe sagging)^9^ were initially recruited. Eligible patients were male or female who were willing to withhold additional aesthetic therapies to the proposed treatment area and were willing to provide informed consent and sign photographic releases. Patients who had been treated with an hyaluronic acid (HA) filler within 1 year; heavy HA fillers, calcium hydroxylapatite (CaHA [Radiesse, Merz North America, Inc., Raleigh, NC]), or poly-l lactic acid (PLLA; Sculptra, Galderma Laboratories, Fort Worth, TX) within 18 months; or PMMA-collagen gel or silicone oil were excluded. Patients with previous tightening procedures in the lower face (within 1 year) or neuromodulator treatments to the platysma (within 6 months) were excluded. Following the initial 1-year study, patients who remained willing to abstain from other treatments and continue with study follow-up visits were recruited. Of the initial 20 patients, 10 remained enrolled in the study.

After obtaining signed informed consent, a negative skin test for bovine collagen allergy within 1 month before treatment, the patient was reevaluated by the investigator using the Merz jawline at rest scale^9^ for the left and right sides of the face before injection and then treated to achieve optimal correction of the lower face and pre-jowl sulcus. Treatment of the post-jowl sulcus, marionette folds, and chin was also permitted. At 4 weeks, a touch-up treatment was permitted if indicated. Additional follow-up visits took place at weeks 12 (n = 19), 26 (n = 17), 52 (n = 18), and 104 (n = 10). For each posttreatment visit, the patient was evaluated by a single-blinded evaluator (advanced registered nurse practitioner) using the Merz jawline at rest scale^9^ to evaluate posttreatment photographs in comparison with baseline photographs for the left and right sides of the lower face. At each posttreatment visit, patients were also evaluated by the treating physician using the Physician-reported Global Aesthetic Improvement Scale (PGAIS), and the patient evaluated their improvement using the Subject-reported Global Aesthetic Improvement Scale (SGAIS). Both the PGAIS and SGAIS are 5-point scales where 1 = much worse; 2 = worse; 3 = no change; 4 = improved; and 5 = much improved. Efficacy measures were obtained before treatment if the patient received a touch-up treatment at week 4. At each posttreatment time point, patients were asked to rate their satisfaction with their treatment outcome on a scale of 1-6 (1 = very dissatisfied; 2 = dissatisfied; 3 = neither satisfied nor dissatisfied; 4 = somewhat satisfied; 5 = satisfied; and 6 = very satisfied). Patients were compensated for their time and participation in follow-up appointments at weeks 12, 26, 52, and 104. PMMA-collagen gel for this study was provided by the manufacturer (Suneva Medical, Inc., San Diego, CA). Patients were monitored throughout the study for adverse events (AEs). All data were collected in accordance with the Declaration of Helsinki, and the study protocol was approved by US IRB, Miami.

### Endpoints and Statistical Analysis

The primary endpoint was a 1-point improvement on the Merz Aesthetics Scale for jawline at rest. Using each jawline as a separate data point, a 2-sided paired *t*-test was used to assess statistical significance for change in the Merz jawline score at weeks 4, 12, 26, 52, and 104 compared with baseline. Satisfaction scores, PGAIS scores, and SGAIS scores are presented using descriptive statistics.

## RESULTS

### Demographics

The study took place between November 2018 and July 2021. The study required 6 weeks for the skin test, took time to completely enroll, and included 2 years of follow-up. The study enrolled 18 female and 2 male patients with a mean age of 61.7 (48-73) years and jawline severity scores of 2-4 (0 = no sagging, 1 = mild sagging, 2 = moderate sagging, 3 = severe sagging, and 4 = very severe sagging). All patients were treated using the serial column technique with a 27-G, 0.5-inch needle in the subdermal/subcutaneous plane, primarily in the pre-jowl sulcus, but also in the post-jowl sulcus, marionette folds, and/or chin when treatment was needed to obtain a balanced aesthetic effect. For a single injection with this injection technique, the needle is placed into the deep subcutaneous plane in a perpendicular fashion, and ~0.05 mL of filler is injected in a retrograde fashion until the needle reaches the subdermal plane. The mean volume injected per patient (for both sides) for initial treatment was 1.66 mL and was 1.46 mL for touch-up treatment, for a total mean volume per patient of 3.11 mL. Jawline severity scores were collected for all 20 patients on day 0 and week 4, with 1 patient withdrawing due to work schedule at week 12, another withdrawing to undergo a neck lift at week 26, and one additional patient withdrawing at week 52 due to concerns surrounding COVID-19. A single patient missed the week-26 follow-up but returned for the week-52 follow-up visit. For 7 of the 17 patients who were evaluated at 52 weeks, the final evaluation was outside of the established follow-up window in the protocol due to mandatory office closures prompted by COVID-19. These 7 patients were evaluated in person between 58 and 61 weeks.

A total of 10 patients, including 8 females and 2 males, participated in an extended follow-up visit at approximately week 104. For patients present at the final evaluation the mean follow-up was 107 weeks (range 2 years [104 weeks] to 2.3 years [121 weeks]). Ten (50%) patients who were present at the start of the study (week 0) did not participate in the extended 104-week follow-up visit. Among those patients, 3 of them did not respond to the request for a follow-up visit; 3 patients elected to stay home due to the COVID-19 pandemic; 3 patients were ineligible due to thread lifts; 1 patient had a neck lift, also making them ineligible for follow-up. None of the patients who withdrew did so because of AEs.

At baseline (week 0), the mean jawline score was 2.4 (range 2.0-3.0) for the left side of the face and 2.45 (range 2.0-4.0) for the right side of the face. The average total volume of PMMA-collagen gel (including initial volume and touch-up) injected for both sides of the face was 3.11 (range 1.8-4.7). All 20 patients received a “touch-up treatment” at week 4.

### Improvement in Jawline Score

**Figure 1.**
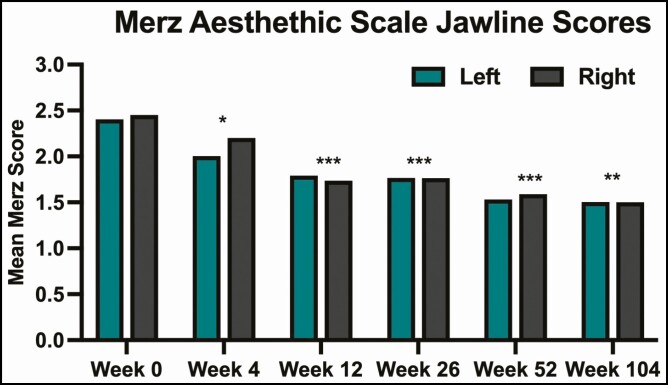
Mean scores on the Merz Aesthetic Scale for jawline at rest (0 = no sagging, 1 = mild sagging, 2 = moderate sagging, 3 = severe sagging, and 4 = very severe sagging).^9^ Asterisks indicate significant differences compared with week 0 as determined by a 2-tailed *t*-test. *P < 0.05; **P < 0.01; ***P < 0.001.

A statistically significant improvement in jawline score for both the left and right sides of the face was observed at all posttreatment visits. At week 4, before touch-up treatment, jawline scores at baseline (2.4 [left], 2.45 [right]) were improved by 0.4 points (range 0-1, *P* = 0.002) on the left and 0.25 points (range 0-1, *P* < 0.02) on the right. At 12 weeks (8 weeks following touch-up), jawline scores were improved by 0.61 points (range 0-1, *P* < 0.001) on the left and 0.71 points (range 0-1, *P* < 0.001) on the right. At week 26 (22 weeks after touch-up), mean jawline scores were improved by 0.64 points (range 0-1, *P* < 0.001) on the left and 0.69 points (range 0-1, *P* < 0.001) on the right. At 52 weeks (48 weeks post-touch-up), the mean improvement in scores was 0.87 points (range 0-2, *P* < 0.001) for the left side of the face and 0.86 points (range 0-1, *P* < 0.001) for the right side. At 104 weeks (100 weeks post-touch-up), mean jawline scores improved by 0.90 points (range 0-2, *P* < 0.01) for the left side and 0.95 points for the right side (range 0-2, *P* < 0.01) (Figure 1). At all post-touch-up treatment time points, the mean improvement in jawline score was <1 (range 0.61 at week 12 to 0.90 at week 104 for the left side; range 0.71 at week 12 to 0.95 at week 104 for the right side).

### Physician and Subject GAIS

**Figure 2.**
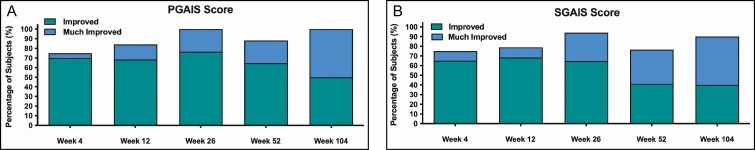
(A) Physician Global Aesthetic Improvement Scale scores (PGAIS) and (B) Subject Global Aesthetic Improvement Scale scores (SGAIS).

At week 4, before administration of the touch-up treatment, PGAIS scores were at least “improved” for 75% of patients, with the remainder of patients rated as having “no change” (Figure 2A). However, following touch-up treatment at weeks 12, 26, 52, and 104, the percentage of patients who were rated as “improved” or “much improved” increased to 84%, 100%, 88%, and 100%, respectively. At each time point, the majority of patients were rated as at least “improved” according to the PGAIS. At week 4 before the touch-up injection, SGAIS scores for appearance were at least “improved” for 75% of patients (Figure 2B). At weeks 12, 26, 52, and 104, the percentages of patients who felt that their appearance was at least “improved” were 79%, 94%, 76%, and 90%, respectively. Similar to the PGAIS score, most patients felt that their appearance was at least “improved” at each time point. At week 4 before touch-up treatment, 75% of patients rated their appearance at least “improved,” whereas 25% rated their appearance as “no change.”

### Patient Satisfaction

**Figure 3.**
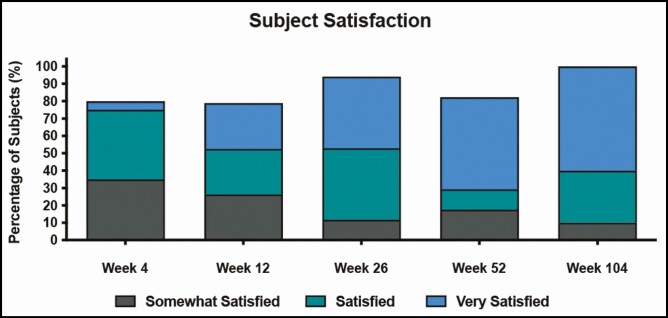
Subject satisfaction with treatment outcome.

At all posttreatment time points, a majority of patients were satisfied with their treatment outcome. At 4 weeks, 80% of patients were at least “somewhat satisfied.” At weeks 12 and 26, 79% and 94% of patients were at least somewhat satisfied, respectively. This satisfaction persisted to the end of the study when, at 52 and 104 weeks, the percentage of patients at least “somewhat satisfied” was 82% and 100%, respectively. The proportion of patients who were highly satisfied increased throughout the study, with 26%, 41%, 53%, and 60% of patients indicating that they were “very satisfied” at weeks 12, 26, 52, and 104, respectively (Figure 3).

### Safety Analysis

Throughout the study, patients were monitored for AEs. A total of 4 patients experienced a total of 6 incidents of injection-site bruising. One patient who experienced mild post-injection bruising at baseline and follow-up did not require treatment, but 3 patients with moderate bruising were treated with the V-beam pulsed-dye laser at week 4 or week 12 to expedite resolution (1 patient was treated at both weeks 4 and 12). No patients had unresolved bruising at week 26. Two patients had small, 2- to 3-mm, nodules along the pre-jowl area. These small nodules were not visible, red, inflamed, or tender, and appeared along the jawline in both patients. The nodules appeared at week 26 in 1 patient and at week 12 in the other patient. For both patients, the nodules resolved by the next study visit and did not require treatment. No AEs were reported for any patients at weeks 52 or 104.

### Representative Patient Images

**Figure 4.**
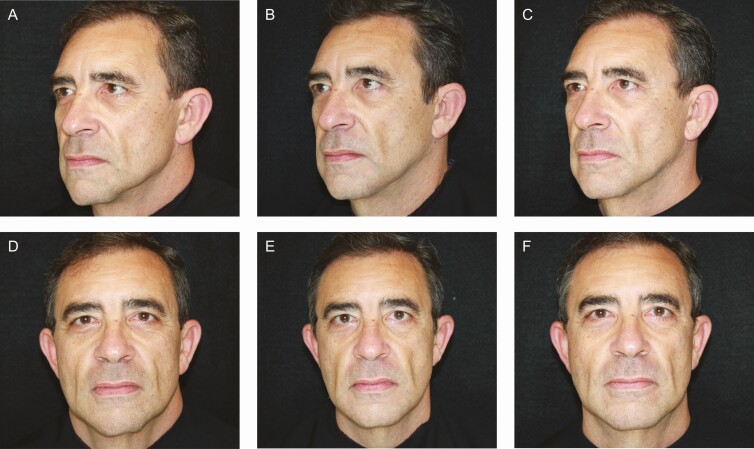
A 60-year-old male injected with PMMA collagen gel in the prejowl sulcus is shown at baseline (A, D) and at 52 weeks (B, E) and 104 weeks (C, F) posttreatment.

**Figure 5.**
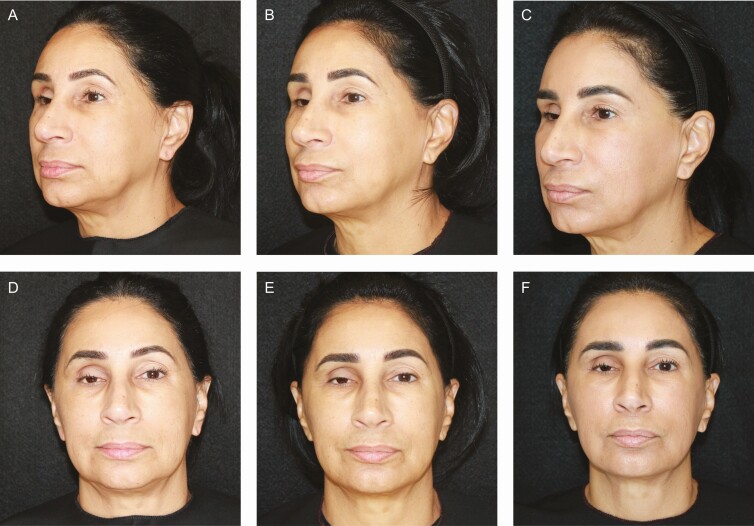
A 59-year-old female injected with PMMA collagen gel in the prejowl sulcus is shown at baseline (A, D) and at 52 weeks (B, E) and 117 weeks (C, F) posttreatment.

**Figure 6.**
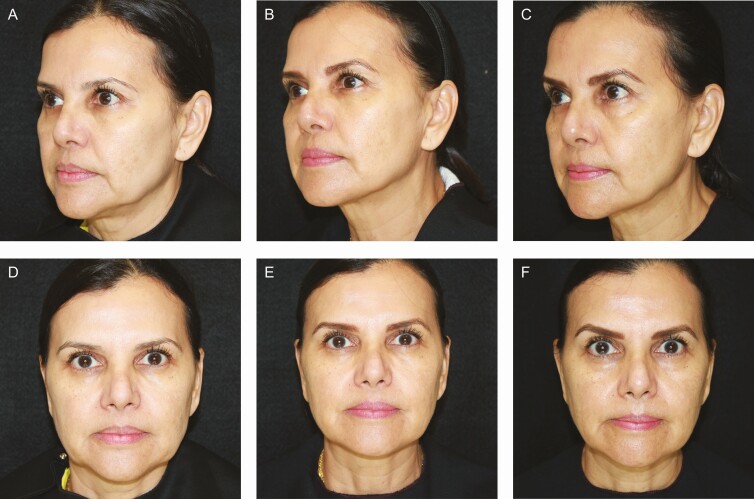
A 55-year-old female injected with PMMA collagen gel in the prejowl sulcus is shown at baseline (A, D) and at 52 weeks (B, E) and 121 weeks (C, F) posttreatment.

Figures 4-6 provide examples of the results achieved in this study.

## DISCUSSION

In this study, treatment of the pre-jowl sulcus with PMMA-collagen gel resulted in a significant, durable improvement for the extent of 104 weeks in the appearance of sagging along the jawline. At 12 weeks (8 weeks post-touch-up), the mean score on the Merz Aesthetic Scale for jawline at rest improved from 2.4 and 2.45 on the left and right sides of the face, respectively, to 1.79 and 1.74 on the left and right sides of the face (*P <* 0.001 for both sides). Improvement persisted at 52 weeks, with mean scores for the left and right sides of the face of 1.53 and 1.59, respectively (*P <* 0.001 for both sides; Figure 1). This progressive improvement in severity scores over time is indicative of the ongoing biostimulatory effect of PMMA.^4^ Because most of the patients enrolled in this study had baseline scores of 2 (moderate sagging, 60% [12/20] left side and 60% [12/20] right side), a single point in improvement would correspond to a transition to mild sagging for most patients. Thus, one potential shortcoming of the study is that intermediate improvements may not be adequately captured by a 5-point scale. In this study, the mean change in jawline score on the Merz Aesthetics Scale was slightly <1 at all posttreatment time points, but 94% of patients rated themselves as at least “improved” on the SGAIS and 94% of patients indicated that they were at least “somewhat satisfied” at week 26. This inconsistency between objective scale measures of the jawline and patient perceptions of global improvement is consistent across all time points. However, the mean change in jawline score approached the primary endpoint at week 104, with 0.90 and 0.95 improvement points for the left and ride sides of the face, respectively. Moreover, at 104 weeks, 50% of patients rated their appearance as “much improved” and 40% of patients rated their appearance as “improved.” Taken together with the 100% of patients who were rated as at least “improved,” by the investigator (Figure 2) and the 100% of patients who were at least “somewhat satisfied” with their results at 104 weeks (Figure 3), these findings support the assertion that treatment with PMMA-collagen gel results in substantial and durable improvement. This finding is consistent with clinical studies of PMMA-collagen gel, showing ongoing satisfaction at 5 years (84% at least “satisfied”) following the treatment of the nasolabial folds.^5^ Importantly, as treatment of the post-jowl sulcus, marionette folds, and chin was permitted, satisfaction could be driven by a more global change in lower-face appearance. The inclusion of multiple treatment areas is a confounder.

One possible contributor to the relatively modest change in jawline score observed in comparison with documented effects in the nasolabial folds and midface is the issue of non-response.^8^ In clinical practice, a small percentage of patients simply do not respond to revolumization of the pre-jowl sulcus as expected. In these patients, additional techniques such as lift with absorbable suspension sutures may be used, or surgery may be required. It is impossible to identify these nonresponders before treatment. In this study, 1 patient had no change in jawline score on either the left or right side through week 52 (this patient did not participate in the 104-week follow-up visit). This nonresponder had baseline scores of 2 and 3 for the left and right sides, respectively, and was not the most severe patient treated. The sample size of this study is too small to determine the percentage of patients who can be expected not to respond to treatment or identify the features of patients who are nonresponders, a limitation, but this may be related to the collagen-producing capacity of the skin. In addition to this 1 patient, 4 other patients had a 1-point change on one side and no reported change on the other side by week 52; however, most patients (n = 15) had at least a 1-point change on one or both sides. At 104 weeks, 70% of patients (n = 7) had at least a 1-point change from baseline on both sides of the face. The remaining 30% of patients (n = 3) had no difference on both sides at week 104 compared with baseline jawline scores; however, those 3 patients felt that their appearance had either “improved” or “much improved” and they were either “satisfied” or “very satisfied” with treatment. The use of a single-blinded reviewer, rather than multiple reviewers, is also a limitation of the study.

While there are multiple fillers that may be used in clinical practice (including HA fillers), the durability of PMMA-collagen gel makes it well suited for the treatment of the lower face, especially for the pre-jowl sulcus. PMMA microspheres are not absorbed by the body, and the ongoing collagen stimulation promoted by the microspheres gives rise to a highly durable effect.^4^ In clinical studies, the activity of PMMA-collagen gel has been maintained through the end of the 5-year observation period, and benefit persisted at the end of this 2-year study.^5,10^ This high durability is desirable for applications for which the effect is expected to persist for several years, including treatment of acne scars, correction of depressions on the bridge of the nose,^11^ or even creation of a basic platform for rejuvenation to prevent patient return to baseline. Indeed, studies have shown that treatment of atrophic acne scars using PMMA-collagen gel is effective (91%), with high patient satisfaction (84%).^7,12^ In the current study, patients were treated in the pre-jowl sulcus, an area where durable improvement of the patient’s personal baseline is desired. Due to the durability of treatment and irreversibility of injection, the author (O.H.) recommends that injection be performed by advanced physician injectors only and be spread out across multiple visits. Here, all patients received touch-up treatments at week 4: clinically, when working with PMMA-collagen gel, conservative placement of the filler allows for careful correction, and placement of the filler over multiple visits until optimal correction is reached is acceptable.

Of note, there were 2 patients who developed small, non-inflammatory nodules following injection. These small nodules, or lumps, appeared along the pre-jowl mandible and dissipated without intervention by the next study visit. No patients reported AEs at 104 weeks, and there were no granulomas reported at that time. This is a comparatively small study, but the absence of granulomas is consistent with the findings in the PMMA-collagen gel 5-year study where, in 1008 patients, the rate of granuloma was 1.7% (n = 17).^5^ In the 5-year study, the diagnosis of granuloma was confirmed through biopsy; thus, it may not include transient nodules like those reported here. In another 12-year post-approval study of PMMA-collagen gel in 391 patients, lumpiness was reported in 3.3% of patients,^13^ but it is unclear if the lumpiness reported has the same transient characteristics of the ~2-mm nodules observed in this study. While this study is somewhat limited by its size and duration of follow-up, given the longevity of PMMA, results indicate that PMMA-collagen gel is an effective, long-lasting modality for the treatment of the pre-jowl sulcus and represents a unique tool in the aesthetic physician’s toolbox for treatment of the aging face.

## CONCLUSIONS

PMMA-collagen gel may be used to rejuvenate the lower face by treating the pre-jowl sulcus and define the jawline. In this study, there were no late-emergent AEs, and patient satisfaction with the procedure was high.
